# Research progress in the treatment of chronic fatigue syndrome through interventions targeting the hypothalamus-pituitary-adrenal axis

**DOI:** 10.3389/fendo.2024.1373748

**Published:** 2024-04-10

**Authors:** Yi-Dan Zhang, Li-Na Wang

**Affiliations:** ^1^ College of Basic Medicine, Naval Medical University, Shanghai, China; ^2^ Department of Traditional Chinese Medicine, Naval Medical University, Shanghai, China

**Keywords:** chronic fatigue syndrome, hypothalamus-pituitary-adrenal axis, dysfunction, modern medicine, traditional Chinese medicine

## Abstract

Chronic fatigue syndrome (CFS) causes great harm to individuals and society. Elucidating the pathogenesis of CFS and developing safe and effective treatments are urgently needed. This paper reviews the functional changes in the hypothalamus-pituitary-adrenal (HPA) axis in patients with CFS and the associated neuroendocrine mechanisms. Despite some controversy, the current mainstream research evidence indicates that CFS patients have mild hypocortisolism, weakened daily variation in cortisol, a weakened response to the HPA axis, and an increase in negative feedback of the HPA axis. The relationship between dysfunction of the HPA axis and the typical symptoms of CFS are discussed, and the current treatment methods are reviewed.

## Introduction

The term chronic fatigue syndrome (CFS) was first proposed by the U.S. Centers for Disease Control and Prevention in 1988 (1). To improve the definition and diagnostic criteria, the International CFS Study Group released two revisions in 1994 and 2003 (2, 3) and gradually established the widely used “gold standard”; that is, CFS is a syndrome characterized by chronic fatigue that is clinically assessed, unexplained, persistent or recurrent, new or with a definite onset, non-congenital, not due to ongoing labor, and not relieved. Additionally, the occupational ability, educational ability, social ability and personal life of affected individuals are substantially worse than those before the illness. For the diagnosis of CFS, four or more of the following symptoms persist or recur for at least 6 months, the appearance of which does not occur prior to fatigue symptoms: ① severe impairment of short-term memory and concentration, causing a substantial decrease in occupational ability, educational ability, social ability and personal life compared with those before disease onset; ② sore throat and tenderness of the neck or axillary lymph nodes; ③ muscle pain and multiple joint pain not accompanied by swelling; ④ type, manner, and severity of headache attacks different from those before; ⑤ inability to recuperate after sleep; ⑥ and persistent fatigue for more than 24 hours after activity. It is estimated that 836,000 to 2.5 million people are affected with CFS in the U.S., with as many as a quarter being homebound or bedridden (4). However, considering the number of people affected and the harm caused, CFS has not received due research attention (5). Notably, in 2018, an article in “Nature” called for the reinitiation of CFS studies (6).

Due to the enormous complexity of CFS, its pathogenesis is still unclear. Some of the researchers suggest immunological, neuroendocrine and metabolic pathways that causes CFS, which indicates significant immune dysregulation (7, 8). Neuroendocrine mechanisms have been the focus of some research. In 1991, several researchers focused on the hypothalamus-pituitary-adrenal (HPA) axis (9), and since then, the HPA axis has been a research hotspot in the field of neuroendocrine mechanisms. The HPA axis has three levels: the hypothalamus is the first level, and corticotropin releasing hormone (CRH) secreted by the hypothalamus activates the HPA axis; the pituitary gland is the second level, and CRH can promote the secretion of adrenocorticotropic hormone (ACTH) from the pituitary gland; and the adrenal gland acts as the third level and is regulated by ACTH, cortisol (CORT) secreted by the adrenal gland is the main functional product of the HPA axis. Additionally, CORT acts on the hypothalamus and the pituitary gland through a negative feedback mechanism, which inhibits the secretion of CRH and ACTH to stabilize CORT levels (10). The aim of this paper was to investigate the functional changes and impact of the HPA axis in patients with CFS and to review the current treatment methods.

## HPA axis dysfunction in CFS patients

The plasma CORT levels in CFS patients were first reported by a researcher in 1981 (11). Since then, clinical factors have been gradually expanded to include urine CORT (12) and saliva CORT (13); additionally, because CORT secretion has diurnal variation and is pulsatile, observation times have also gradually changed from a single time point (14) to overall circadian rhythm (15).

**Figure 1:**
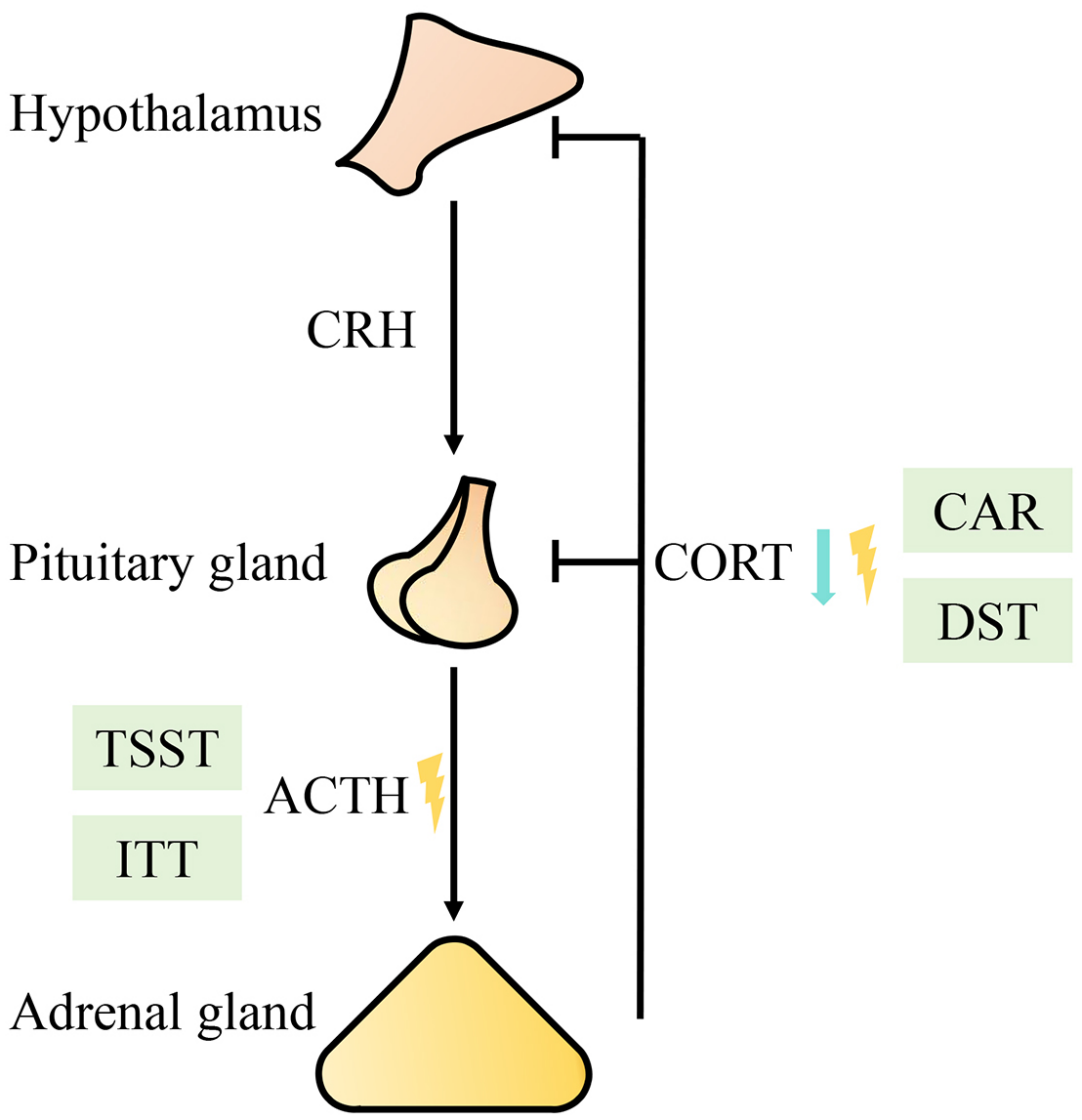
Diagram of functional changes in the HPA axis. The blue arrow represents a decreased level; the yellow lightning bolt represents a rhythm disorder. Abbreviations: CRH (corticotropin releasing hormone), ACTH (adrenocorticotropic hormone), CORT (cortisol), TSST (Trier Social Stress Test), CAR (cortisol awakening response), ITT (insulin tolerance test), DST (dexamethasone suppression test).

Current mainstream research evidence supports that CFS patients have mild hypocortisolism, weakened daily variation in CORT, unresponsiveness of the HPA axis, and enhanced negative feedback from the HPA axis (16). Several studies have shown that, compared with those in healthy controls, the plasma (14, 17, 18), urine (12), and saliva (19) concentrations of CORT in CFS patients are significantly lower. In addition to observations at a single time point, changes in the circadian rhythm of the HPA axis have also been analyzed. Through the collection and comparison of saliva CORT concentrations at multiple time points in 24 hours, it was found that the CORT levels in CFS patients were lower in the morning and higher in the evening; that is, daily CORT variability was attenuated compared with that in normal controls (20). More intensive plasma collection revealed that the secretion rhythm of ACTH in CFS patients was significantly different from that in normal controls, with release decreasing during the physiological morning peak (21). These findings demonstrated an alteration in the secretion rhythm of the HPA axis. Using the Trier Social Stress Test (TSST), researchers found that, compared with healthy controls, CFS patients had a significantly reduced area under the ACTH response curve, with no significant difference in the area under the CORT response curve (22). Cortisol awakening response (CAR) was tested, and it was found that, compared with healthy controls, CFS patients with childhood trauma experienced a significantly reduced area under the CORT response curve and that their CORT response curves were flatter (23). In addition, women with CFS had significantly lower CAR levels than healthy women did, and the increase in the area under the response curve was also lower (24). An insulin tolerance test (ITT) showed that, in CFS patients, the area under the ACTH response curve was significantly reduced, while that of CORT was not significantly different from that for controls (25, 26). This finding confirmed that there was a blunted response of the HPA axis. The enhancement of negative HPA axis feedback has been tested using the dexamethasone suppression test (DST). After the administration of 0.5 mg dexamethasone, the saliva CORT output was significantly lower in CFS patients than in healthy controls (27),the saliva CORT response significantly decreased (28). After the administration of 5 mg of prednisolone, the saliva CORT levels and urine CORT metabolite levels were significantly lower in CFS patients than in individuals in the control group, and the percentage of inhibition was significantly higher in CFS patients than in individuals in the control group (29), demonstrating the enhancement of negative feedback on the HPA axis (**Figure 1**).

However, not all the study results are consistent with the mainstream conclusions. Several studies have shown that there is no significant difference between the CORT levels in CFS patients and those in healthy controls (30). Using the TSST, researchers found that childhood traumatic experience was not significantly associated with CORT output (31). There were no significant differences between a CFS group and a healthy control group in terms of CAR, daily CORT secretion curve, or CAR after DST (32). The differences between these studies may be explained by the study design and insufficient sample size. However, the mainstream conclusions still need verification through comprehensive studies with larger sample sizes (33).

## The effects of HPA axis dysfunction in CFS patients

Because most studies tend to analyze patients who have been ill for many years, it is not clear whether HPA axis dysfunction is the cause or result of CFS (16). Therefore, some researchers have proposed that the changes in the HPA axis in CFS patients may be an incidental phenomenon that occurs later in the disease course rather than having special etiological significance (34). However, considering that HPA axis dysfunction may play an etiological and pathological role in CFS, it is still necessary to investigate the association of the HPA axis with typical symptoms of CFS (35).

Dysfunction of the HPA axis, especially hypocortisolemia, is often closely related to fatigue, pain, and increased pressure sensitivity. Various mechanism can explain such dysfunction. The release of different hormones may be reduced, thereby reducing the stimulation of receptors for each hormone. This may also be the result of the excessive secretion of a certain hormone, followed by the downregulated expression of the corresponding target receptor, increased sensitivity to the negative feedback of glucocorticoids, or increased relative resistance to CORT (36). Dysfunction of the HPA axis may also be mediated through immune mechanisms, especially HPA axis dysfunction characterized by hypocortisolism, which can weaken the ability of HPA axis hormones to suppress the immune system; therefore, relatively few physiological or psychological stress signals can be transmitted into inflammatory response through activating inflammasomes and the subsequent proinflammatory cytokines (37, 38). Cytokine-mediated inflammation may also explain the widespread pain and pain hypersensitivity in CFS patients (39). In addition, the circadian rhythm of melatonin (MT) secretion, the most important sleep regulation factor, is opposite to that of the HPA axis, and there is an interaction between the two (40). In CFS patients, changes in the circadian rhythm of the HPA axis have been observed; the amplitude of the circadian secretion of CORT is reduced, the peak phase of CORT secretion is advanced, and the peak phase of MT secretion is delayed. Abnormalities in the peak phases of the two disorders can lead to sleep disorders, providing a neuroendocrine explanation for CFS patients’ difficulty falling asleep at night and easy awakening early in the morning (41).

## Treatment of CFS patients with modern medicine

The 2023 updated guidelines of the CDC noted that there is currently no cure or approved treatment for CFS, with the management of symptoms being the only treatment approach. The guidelines recommend treatment for post-exertional malaise, sleep, pain, anxiety, stress, depression, orthostatic intolerance, and memory and attention problems, with an emphasis on activity management (42). Activity management involves patients finding their mental and physical activity limits and then planning their activities and rest to remain within these limits to avoid post-exertional malaise. Limits vary among individuals; therefore, recording activities and symptoms can help patients discover their personal limits, especially in the early stage of the disease. In addition, patients should be reminded not to try to increase activities beyond their limit even though the activity management plan is working well because post-exertional malaise can recur (43).

The largest study on this topic provided favorable evidence for the efficacy and safety of cognitive behavioral therapy and graded exercise therapy (44, 45). However, when the data were re-analyzed, the results changed significantly (46). Subsequent studies have questioned their efficacy and safety (47). After comprehensive consideration, the CDC no longer recommends both for the treatment of CFS. Recently, new randomized controlled trials (RCTs) have been conducted to find more evidence, thus indicating that modern medicine is still making efforts to find a better treatment plan for CFS (48, 49).

Regarding HPA axis dysfunction, randomized controlled trials have shown beneficial effects of hydrocortisone for some patients, but the overall evidence of its efficacy is insufficient (50–54). Long-term treatment is associated with adverse effects, including Cushing’s syndrome, osteoporosis, extreme mood changes and seizures (55); therefore, hydrocortisone was not included in the CDC guidelines.

## Treatment of CFS patients with traditional Chinese medicine

Due to limited treatment options, many CFS patients receive traditional medicine and alternative therapies, including traditional Chinese medicine (TCM) (56). The modern medical term “chronic fatigue syndrome” does not accurately correspond to the TCM literature. According to its clinical symptoms, “CFS” is often categorized as “asthenia” (57). The main characteristics of “fatigue” were first described during the Han Dynasty in one of the most important works of TCM: “Synopsis of the Golden Chamber” by Zhongjing Zhang (58). In summary, the etiology includes the following four aspects: ① damage to five internal organs (including qi, blood, yin, and yang) caused by exogenous invasion; ② fatigue (including physical, mental, and sexual fatigue); ③ emotional imbalance (happiness, anger, worry, obsessive thoughts, sadness, fear, and shock); ④ and improper diet (59). There are many methods used by TCM to treat CFS, including acupuncture, massage, cupping, and Chinese medicine, which can relieve pain and improve quality of life (58).

Although it is still not clear whether HPA axis dysfunction is the cause or an incidental phenomenon of CFS, the HPA axis is clearly a potentially important target for studying treatment strategies (35). **Table 1** lists the 7 RCTs of TCM treatment methods that reported changes in HPA axis indicators (60–66). Six studies reported changes in CORT levels. The treatments included pestle needle therapy combined with electric acupuncture, Fuyang cupping, five-element gongdiao (positive mode) music combined with Lixujieyu decoction, acupoint sticking, Lixujieyu decoction, and electric acupuncture (60–62, 64–66). In a study of CFS patients treated with pestle needle therapy combined with electric acupuncture, the CORT level in patients was lower after treatment than before treatment (60). The remaining 5 studies reported that treatment effectively increased CORT levels (61, 62, 64–66) and increased the circadian secretion amplitude of saliva CORT and restored CORT peak phase that had tended to shift forward (66). Three studies reported changes in ACTH levels. The treatments included pestle needle therapy combined with electric acupuncture, scalp acupuncture, and acupoint sticking; all the reported treatment methods effectively increased ACTH levels (60, 63, 64). One study reported changes in MT, and treatment with electric acupuncture increased salivary MT levels and increased the variation in diurnal salivary MT secretion, restoring MT peak phases that had tended to shift backward (66). The occurrence of a CORT regulation exception in these clinical studies has sparked our thinking (60). A number of animal experiments have shown that CFS model rats exhibit hyperfunction of the HPA axis and that after TCM treatment, CORT levels significantly decrease (67–73). A study showed that when the body is subjected to a variety of noxious stimuli, HPA axis function increases, and the release of ACTH and CORT increases, thereby improving tolerance to stimulation and survival. When the regulatory ability is disrupted, HPA axis function is inhibited, adrenal function decreases, and the CORT concentration decreases (74, 75). Therefore, we speculate that the HPA axis has two disordered states, i.e., a hyperactive state and an inhibitory state, and that these states are related to the duration of stress. In most established animal models, the animals are in a state of HPA axis hyperactivity due to short modeling time. The patients in clinical studies are mostly chronically ill and more likely to be in an inhibitory state. In the absence of HPA axis inhibition, TCM treatment methods may not be limited to simply increasing or decreasing the level of a certain hormone but can resolve HPA axis dysfunction. That is, TCM therapeutic approaches have a bi-directional regulatory effect on HPA axis dysfunction in CFS. Although TCM treatment methods provide new possibilities for the treatment of CFS, as suggested by evidence-based research, the relevant RCT studies have deficiencies in terms of experimental design, random allocation concealment, blinding, and safety reporting, thus limiting the interpretation of evidence. Considering these limitations, it is necessary to conduct RCT studies with larger samples and more standardized treatments to provide evidence for the efficacy and safety of TCM treatment methods (76–78).

**Table 1:** RCTs of TCM treatment methods that reported changes in HPA axis indicators.

Study	Experimental group	Control group	Experimental group treatment	Control group treatment	Findings
Liu et al. (58)	41 individuals with CFS	41 individuals with CFS	Pestle needle therapy at Hechelu Electric acupuncture at Baihui, Qihai, Guanyuan, Ganshu, Shenshu, Pishu, and Zusanli	Electric acupuncture at Baihui, Qihai, Guanyuan, Ganshu, Shenshu, Pishu, and Zusanli	The ACTH levels in the two groups were significantly higher after treatment than before treatment The CORT levels in the two groups were significantly lower after treatment than before treatment, with the post-treatment CORT level significantly lower in the experimental group than in the control group
Li et al. (59)	30 individuals with CFS	30 individuals with CFS	Treatment with Fuyang cupping on Beishu functional belt	Massage, chiropracty, and cupping	The serum CORT levels in the two groups were significantly higher after treatment than before treatment, with the post-treatment CORT level significantly higher in the experimental group than in the control group
Cai et al. (60)	30 individuals with CFS	30 individuals with CFS (×3 groups)	Oral treatment using Lixujieyu decoction combined with five-element gongdiao (positive mode) music	Oral treatment using Lixujieyu decoction (Chinese medicine group) Five-element gongdiao (positive mode) music (music group) No treatment (blank group)	After treatment, the plasma CORT level in the combination group was significantly lower than that in the Chinese medicine group, music group, and blank group
Gao et al. (61)	36 individuals with CFS	36 individuals with CFS	Scalp acupuncture at Baihui, emotional zone Body acupuncture at Sanyinjiao, Zusanli, and Guanyuan	Body acupuncture at Sanyinjiao, Zusanli, and Guanyuan	The ACTH levels in the two groups were significantly higher after treatment than before treatment, with the post-treatment ACTH level being significantly higher in the experimental group than in the control group
Chen et al. (62)	30 individuals with CFS	30 individuals with CFS	Acupuncture of back-shu acupoints on five internal organs and then traditional Chinese medicine patch (Jiaweisini powder) at back-shu acupoints on five internal organs	Acupuncture of back-shu acupoints on the five internal organs	The serum ACTH and CORT levels in the two groups were significantly higher after treatment than before treatment, and the differences were significantly greater in the experimental group than in the control group
Zhang et al. (63)	33 individuals with CFS	33 individuals with CFS	Lixujieyu decoction with modification according to syndrome	Vicodin, 10 mg 2 times/day Adenosine triphosphate, 20 mg 3 times/day Oryzanol, 20 mg 3 times/day	In the experimental group, serum CORT levels were significantly lower after treatment than before treatment In the control group, serum CORT levels did not change significantly before and after treatment
Zhu et al. (64)	20 individuals with CFS	20 healthy individuals	Electric acupuncture at Zusanli and Shenshu	No therapeutic operation	Before treatment, saliva CORT and saliva MT levels as well as the circadian secretion amplitude of the two hormones were significantly lower in the experimental group than in the control group but increased significantly after treatment Before treatment, the peak phase of saliva CORT secretion in the experimental group tended to be ahead of that in the control group, the peak phase of saliva MT secretion moved backward

## Summary and outlook

Given the negative impacts of CFS on individuals and society, there is an urgent need to elucidate the pathogenesis of CFS and develop safe and effective treatments. In this paper, we reviewed the changes in HPA axis function in CFS patients. Although controversial, the current mainstream research evidence still indicates that CFS patients have mild hypocortisolism, weakened diurnal variations in CORT, unresponsiveness of the HPA axis, and enhanced negative feedback from the HPA axis. We also discussed the association between HPA axis dysfunction and typical symptoms of CFS. Finally, the current treatment methods were reviewed. Due to the lack of modern medical treatments approved for CFS at this time, TCM treatment methods have the potential to become a new treatment strategy; in particular, TCM may have a two-way regulatory effect and resolve HPA axis dysfunction. However, due to limited evidence, it is necessary to conduct RCTs with larger sample sizes and more standardized protocols to provide additional data.

## Author contributions

YZ: Writing – original draft, Writing – review & editing. LW: Writing – original draft, Writing – review & editing.
